# Inhibition of HIF-1α by PX-478 enhances the anti-tumor effect of gemcitabine by inducing immunogenic cell death in pancreatic ductal adenocarcinoma

**DOI:** 10.18632/oncotarget.2948

**Published:** 2014-12-10

**Authors:** Tiansuo Zhao, He Ren, Li Jia, Jing Chen, Wen Xin, Fan Yan, Jing Li, Xiuchao Wang, Song Gao, Dong Qian, Chongbiao Huang, Jihui Hao

**Affiliations:** ^1^ Tianjin Medical University Cancer Institute and Hospital, National Clinical Research Center for Cancer, Key Laboratory of Cancer Prevention and Therapy, Department of Pancreatic Cancer, Tianjin, China; ^2^ Centre for Haemato-Oncology, Barts Cancer Institute, Queen Mary University of London, London, UK

**Keywords:** Pancreatic ductal adenocarcinoma (PDAC), Immunogenic cell death (ICD), PX-478, Gemcitabine (Gem)

## Abstract

Pancreatic ductal adenocarcinoma (PDAC) is the worst prognoses among all the malignancies. Now, gemcitabine (Gem) is the first line chemotherapeutic drug for advanced pancreatic cancer. However, Gem is usually ineffective to the PDAC because of high degree of drug resistance. Hypoxia and immune suppressive milieu are the best-described hallmarks of PDAC; therefore, we investigated the impact of hypoxia inducible factor-1 (HIF-1) inhibitor, PX-478, in combination with Gem on the induction of immunogenic cell death (ICD). We verified that combined treatment with Gem/PX-478 significantly enhanced the anti-tumor effect and increased proportion of tumor infiltrating T-lymphocytes in Panc02-bearing immune-competent but not in immune-deficient mice. Vaccination using Panc02 cell line treated with single agent or in combination showed significant anti-tumor effects. Pancreatic cell lines treated with Gem and PX-478 can induce an increase in eIF2α phosphorylation was correlated with down-regulation of HIF-1α and elicited exposure of CRT and release of HMGB1 and ATP. Only co-treated cells induced DC maturation/phagocytosis and IFN-γ secretion by cytotoxic T lymphocytes. Altogether, combined treatment with Gem/PX-478 showed significantly inhibition on tumor growth and anti-tumor immunization. We propose that inhibition HIF-1α elicits Gem-induced immune response and eliminates PDAC cells by inducing ICD.

## INTRODUCTION

Pancreatic ductal adenocarcinoma (PDAC) is one of the most aggressive human neoplasms, with an overall 5-year survival rate of less than 5% [1]. Even the patients obtained radical resection and adjuvant chemotherapy, the 5-year survival rate is between 15 % and 25 % [2]. Compared to other malignancies, pancreatic cancer is highly resistance to chemotherapy and targeted therapy [3, 4]. Currently, the standard treatment for advanced pancreatic cancer is single-agent gemcitabine (Gem) [5]. However, the clinical effect of Gem remains modest due to an inherent and acquired chemo-resistance [6]. Therefore, new treatment strategies are urgently needed to improve the prognosis of patients with PDAC.

We have previously demonstrated that hypoxia-inducible factor-1α (HIF-1α) plays crucial roles in the pathogenesis and progression of pancreatic cancer [7, 8]. Initially identified as a critical transcription factor mediating cell adaption to hypoxia [9], it was recently found that HIF-1α is overexpressed in pancreatic cancer even under normoxia condition and levels of HIF-1α are positively correlated with tumor progression, angiogenesis, cell migration and poor prognosis [7, 8, 10, 11]. Therefore, inhibition of HIF-1α expression could be a therapeutic target to control this aggressive disease. Importantly, it was reported that HIF-1α is highly expressed in Gem-resistant pancreatic cancer cells and this is associated with epithelial-mesenchymal transition of cancer cells [12], suggesting that inhibition of HIF-1α may have potential to overcome the resistance of PDAC cells to the treatment with Gem. PX-478 (S-2-amino-3-[4V-N,N,-bis(2-chloroethyl) amino]-phenyl propionic acid N-oxide dihydrochloride) is a specific agent that suppresses constitutive and hypoxia-induced levels of HIF-1α in cancer cells [13, 14]. Recent clinical trials have shown that PX-478 achieved a relatively high disease control ratio in the treatment of advanced cancer and 90% of patients were well tolerated to the reagent (Phase I Trial of PX-478, NCT00522652). Thus, combination of Gem with PX-478 may have a potential strategy to improve the efficacy of Gem therapy in patients with pancreatic cancer.

In order to achieve effective treatment, chemotherapy should improve anti-cancer immunity and alleviate intra-tumoral immunosuppression [15, 16]. Recently, immunogenic cell death (ICD) has been considered as the best way to induce an adaptive immune response and improve the efficacy of anti-cancer treatment [17-19]. ICD is a cell death modality that stimulate an immune response against dead-cell antigens and is featured by (1) the dying cell exposure of damage-associated molecular patterns (DAMPs) including calreticulin (CRT) and/or heat shock proteins (HSPs) in pre- or early apoptotic stages; (2) the pre- or early apoptotic secretion of ATP; and (3) the late apoptotic passive release of non-histone chromatin protein high mobility group box 1 (HMGB1) and possibly of HSP70 and HSP90 [18-20]. DAMPs exposure leads to the specific recognition by antigen presenting cells (APCs), i.e., dendritic cells (DCs) and macrophages, which become mature and prime the tumor specific T and natural killer (NK) cells to react against tumor cell antigens [21, 22].

There is limited information on which chemotherapeutic agents can induce ICD in pancreatic cancer. The capacity of Gem on inducing ICD in PDAC is currently debated [23, 24] and it may require a complementary approach to trigger this process [24]. Numerous reports demonstrated that inhibition of HIF-1α could overcome resistance of tumor cells to chemotherapy [25, 26]. Recently, it has been proposed that inhibition of HIF-1α could be a complementary approach for cancer immunotherapy [27]. However, whether HIF-1α inhibitors could promote ICD has not been defined.

In our own studies, we have found that HIF-1α is overexpressed in patients with PDAC [7, 8]. We hypothesize that inhibition of HIF-1α could enhance the therapeutic efficacy of Gem. In this study, we aimed to compare the efficacies of PX-478 (a specific HIF-1α inhibitor) and chemotherapy drug Gem, as a single agent or in combination, in inducing anti-tumor immune response and immunogenic elimination of PDAC tumor in immune-competent and immune-deficient PDAC-engrafted mice. Gem and/or PX-478-induced ICD markers and immune response, including DC maturation/phagocytosis, and activity of cytotoxic T-cells were determined accordingly *in vitro*.

## RESULTS

### PX-478 enhances the therapeutic efficacy of Gem only in the immune-competent mice models

**Figure 1:**
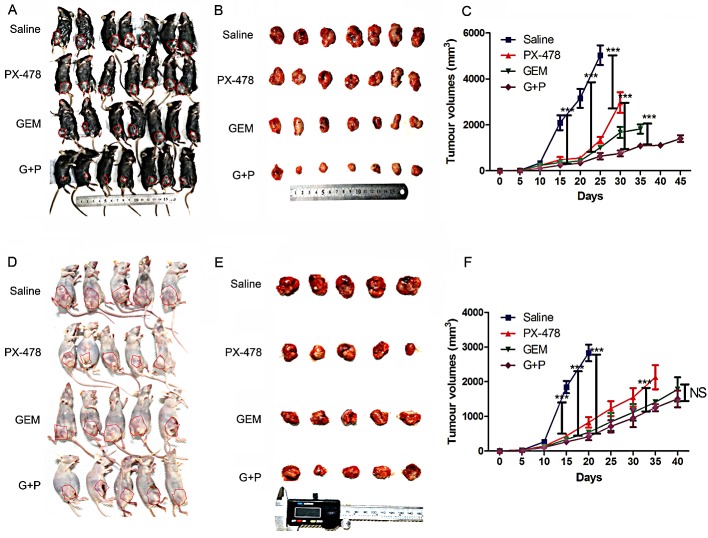
Therapeutic effects of Gem and/or PX-478 in immune-competent and immune-deficient mice Murine PDAC cell line Panc02 was inoculated into the right flank of C57BL/6 mice (7 mice/group) and nude mice (5 mice/group) and subsequently treated with saline (control), Gem (i.p at 15 mg/kg on days 1, 3, 5 every week) and PX-478 (p.o. gavage at 30 mg/kg ×2 consecutive days) and Gem plus PX-478. (A and B) Representative images of tumors formed in C57BL/6 mice. (D and E) Representative images of tumors formed in nude mice. (C and F) Statistical comparisons of tumor volumes in C57BL/6 (C) and nude mice (F). Time-dependent tumor growth of treated groups was analysed by Two-way ANOVA with Bonferroni post-hoc test by comparison with untreated group or Gem/PX-478 treated group compared with single treatment. ***P<0.001. NS indicates no significance.

*In vitro* experiments showed that inhibition of HIF1α by PX-478 sensitized PDAC cells lines to GEM induced apoptosis (data not shown). To evaluate the antineoplastic effects of this combination *in vivo*, we implanted Panc02 cells in immune-competent C57BL/6 and immune-deficient nude mice and treated them with Gem and/or PX-478. Gem or PX-478 alone significantly reduced tumor growth in both immune-competent and incompetent mice (Figure 1) and there was no significant difference when either single treatment efficacy was compared between two mice models. When Gem combined with PX-478, the tumor suppression effect of Gem or PX-478 was significantly increased in immune-competent in C57BL/6 mice (Figure 1A, B and C) but not in nude mice (Figure 1 D, E and F) compared with treated with Gem alone. This suggests that the therapeutic efficacy of this combined treatment with Gem and PX-478 may depend on the presence of thymus-dependent T lymphocytes because it was deficient in nude mice. These results indicate that Gem and PX-478 combination may induce a tumor antigen-specific immune response which in turn elicits elimination of tumor cells.

### Combination of PX-478 with Gem significantly increases the vaccine efficacy compared with single treatment

**Figure 2:**
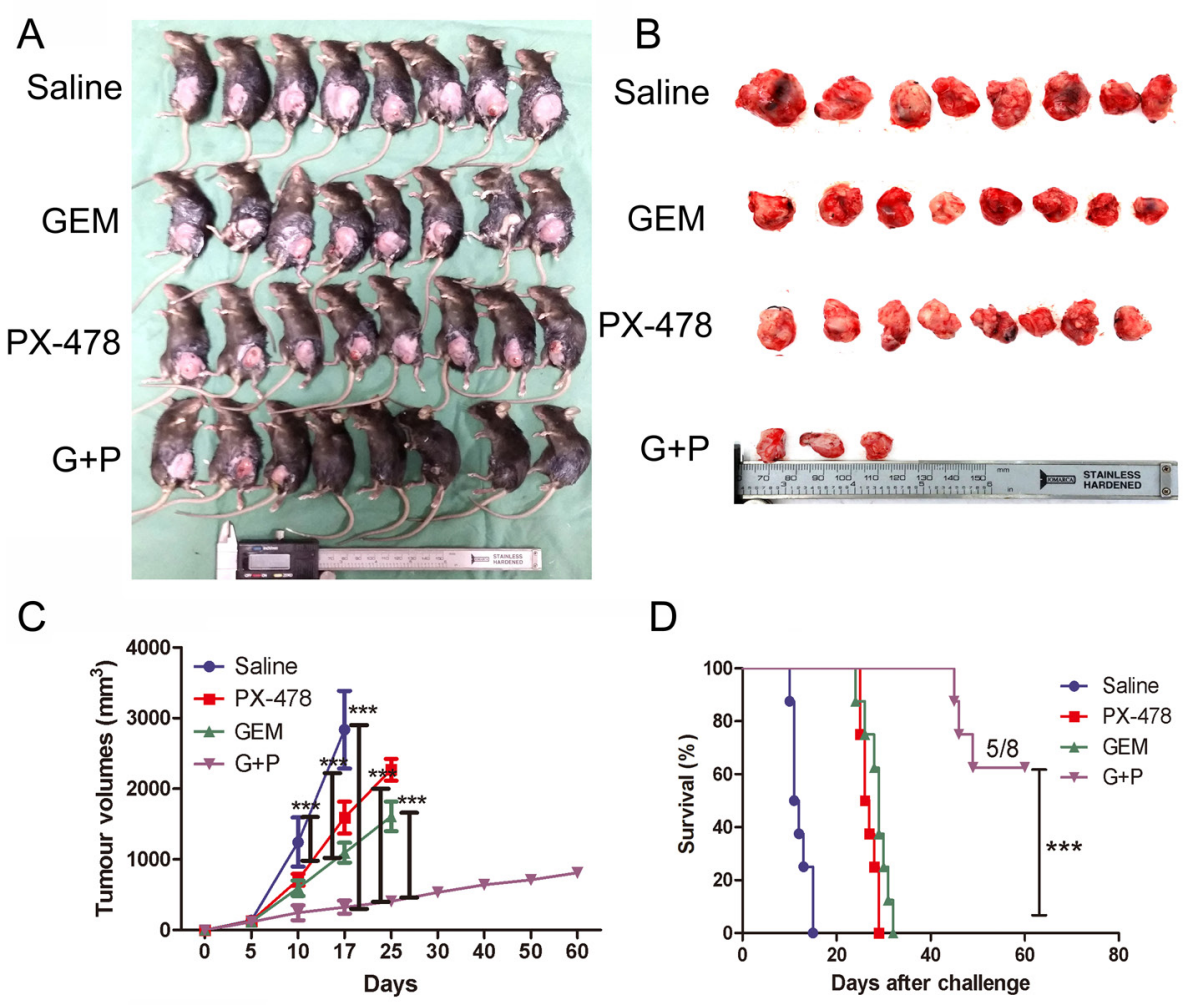
Vaccination impact of Gem/PX-478 on tumor growth in C57BL/6 mice Panc02 cells were *in vitro* incubated with saline, Gem (1.0 μM), PX-478 (25 μM) or both of them for 24 hours. Dying and dead cells/supernatant were subcutaneously injected into C57BL/6 mice (8 mice/group). After 7 days live Panc02 cells were inoculated on the other flank. (A) Image of tumor-bearing mice. (B) Tumors separated from mice. (C) Time-dependent tumor growth. Tumor growth was evaluated by measuring tumor volumes and compared statistically by Two-way ANOVA with Bonferroni post-hoc test. (D) Kaplan-Meier curve of survival rates. Tumor growth was compared using the log-rank test, illustrated with Kaplan–Meier curves. ***P<0.001 indicates comparison of tumor growth between control group with Gem, PX-478 or Gem/PX-478 groups.

To further confirm whether Gem plus PX-478 (Gem/PX-478) combination can elicit immune response, we vaccinated immune-competent C57BL/6 mice with Panc02 cells which were treated with Gem, PX-478 or Gem/PX-478. After one week, the mice were challenged on the other flank with live Panc02 cells. None of the C57BL/6 mice developed tumors on the vaccinated flank. All the mice in the non-immunized group developed tumors at the challenge site and died within 17 days. Either Gem or PX-478-immunized group showed significantly reduced tumor volumes (P<0.001) compared with non-vaccinated group and died within 25 days. Most strikingly, Gem/PX-478-immunized mice showed significantly reduced tumor volumes (P<0.001), and 5 in 8 of mice were alive at 60 days post challenge (Figure 2A, B and C). The survival rate was significantly increased in the Gem/PX-478 group (P<0.001) (Figure 2D). These results demonstrate, for the first time, that Gem/PX-478 combination has high vaccine efficacy against tumor growth via inducing immunogenic cell death.

### Combination of Gem with PX-478 increases infiltrating T cells in tumor-bearing C57BL/6 mice

**Figure 3:**
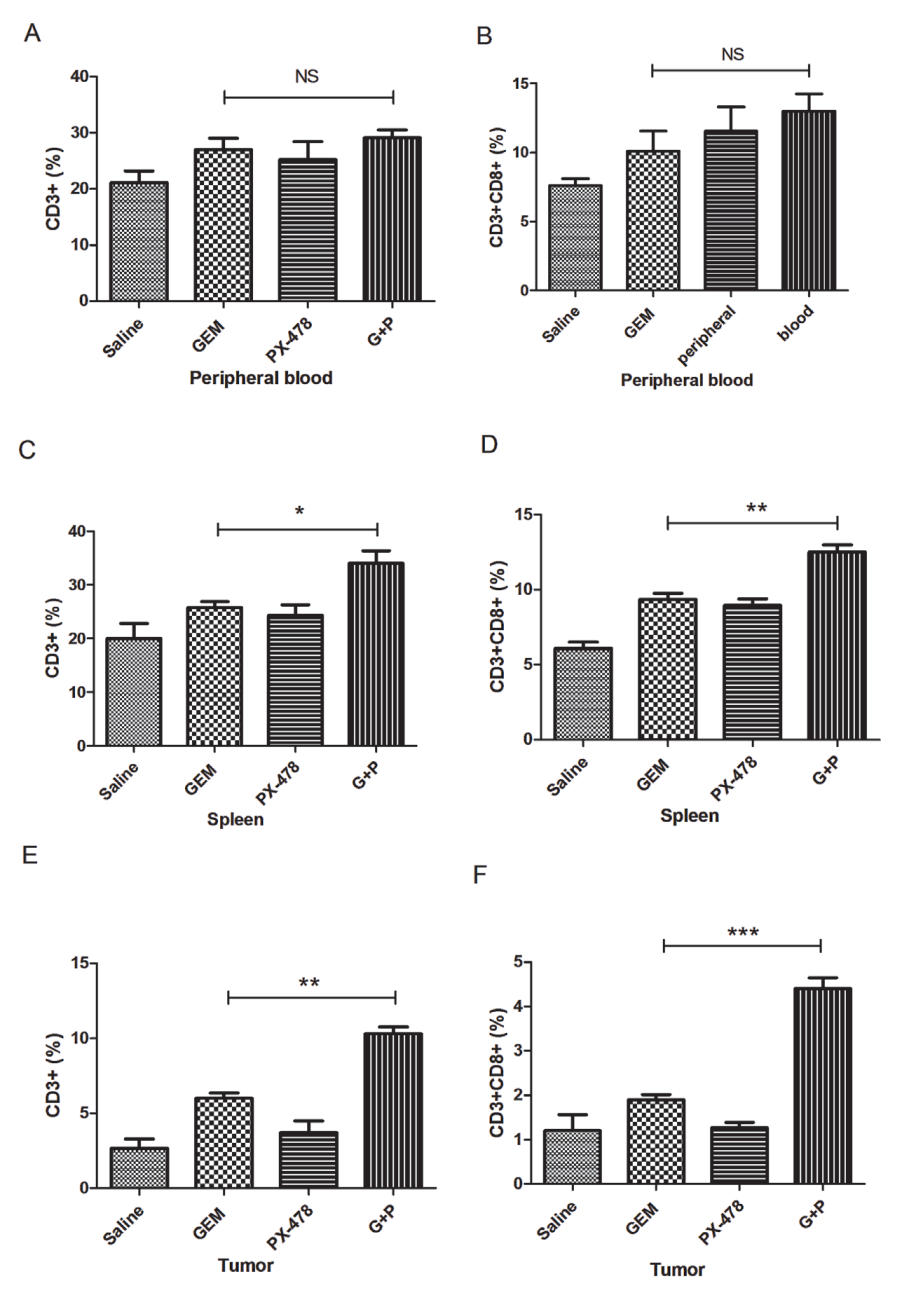
Determination of cytotoxic CD3^+^ and CD8^+^ T lymphocytes Panc02 cells were inoculated into the right flank of C57BL/6 mice (7 mice/group) and subsequently treated with Saline, Gem (i.p at 15 mg/kg on days 1, 3, 5 every week), PX-478 (p.o. gavage at 30 mg/kg ×2 consecutive days), or Gem/PX-478. The proportion of CD3^+^ and CD8^+^ T cells isolated from peripheral blood (A), spleen (B), or tumor (C) were analysed by flow cytometry. Statistical significance was analysed by two-tailed Student's *t*-test. Significantly increased T cells in Gem/PX-478 treated group were compared with treated with Gem or PX-478 alone (n=7). * indicates P<0.05, ** indicates P<0.01 and ‘NS’ means no significance.

We then hypothesised that the pro-survival effect of Gem/PX-478 co-treatment may be due to immunogenic elimination of tumor cells. T lymphocytes from peripheral blood, spleen and tumor of C57BL/6 mice were purified and determined by flow cytometry. The proportions of CD3^+^ and CD8^+^ cytotoxic T lymphocytes in Gem/PX-478 group were not significantly increased in peripheral blood compared with treated with either Gem or PX-478 group (Figure 3A and B). However, significantly increased cytotoxic CD3+/CD8^+^ T lymphocytes were detected in spleen (Figure 3C and D) and tumor tissues (Figure 3E and F) in mice treated with Gem/PX-478 compared with single treatment (Figure 3 and Suppl Figure 1). Collectively, the data suggest that chemotherapy with Gem/PX-478 may eliminate tumor cells by tumor-infiltrating cytotoxic T lymphocytes-mediated ICD.

### Inhibition of HIF-1α sensitizes PDAC cells to Gem or OXP-induced phosphorylation of eIF2α

**Figure 4:**
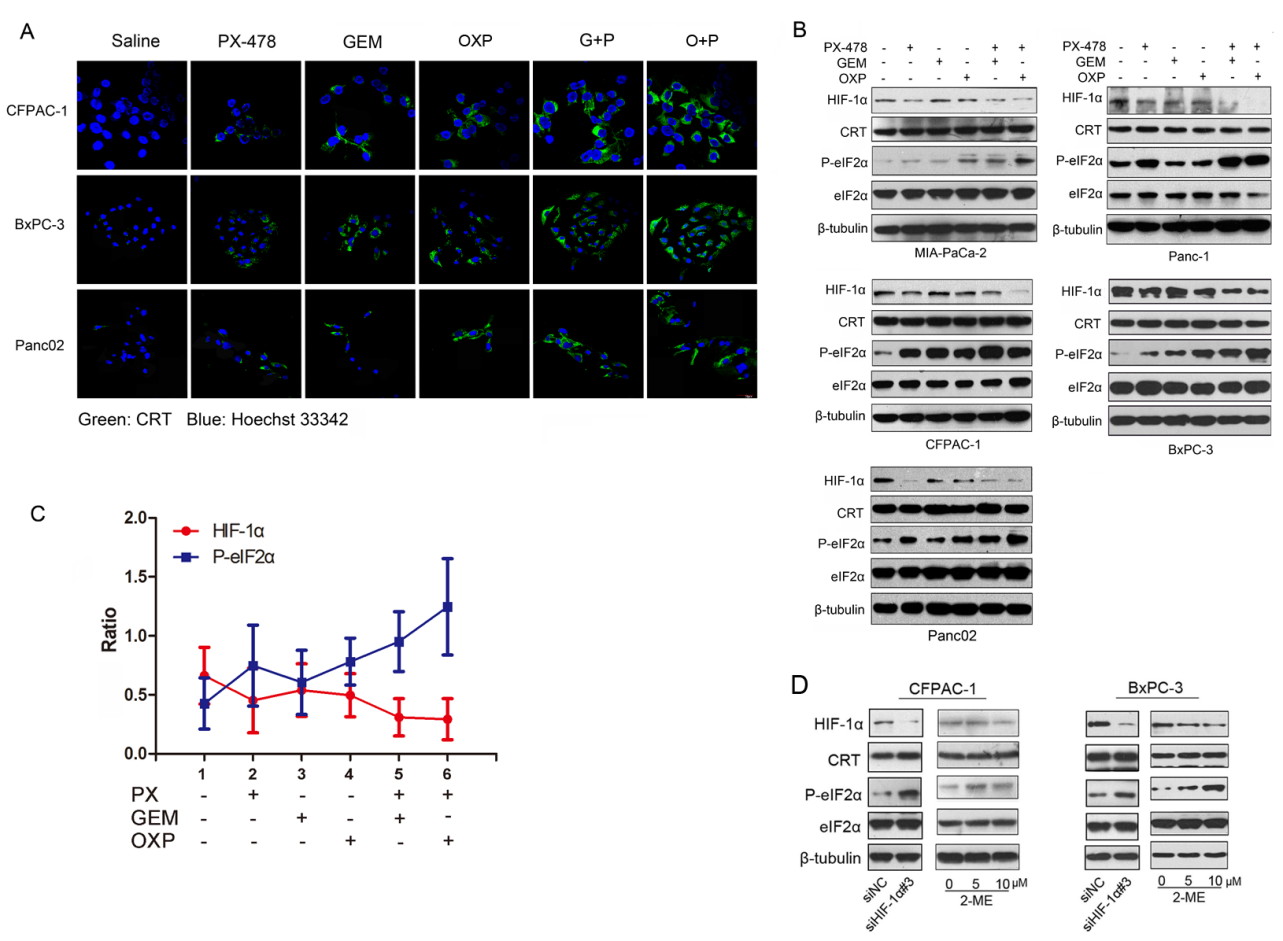
Negative correlation between HIF-1α expression and eIF2α phosphorylation PDAC cell lines were incubated with saline, Gem (1.0 μM), PX-478 (25 μM), Gem/PX-478, OXP (oxaliplatin) (300μM) or OXP/PX-478 for 24 hours. (A) CRT surface exposure on CFPAC-1, BxPC-3 and Panc02 cell lines. Non-permeabilized cells were co-stained with anti-CRT antibody and Hoechst 33342 and determined by confocal microscopy (magnification, 600×). Green colour indicates ecto-CRT. (B) Five PDAC cell lines were treated with GEM, PX-478, OXP (300μM), Gem/PX-478, or Gem/OXP for 24 hours. Expression of HIF-1α, CRT, P-eIF2α, and eIF2α was determined by Western blotting. β-tubulin was used as a loading control and OXP was served as a positive control. (C) Negative correlation between levels of HIF-1α and P-eIF2α. Expression levels of both HIF-1α and P-eIF2α were analysed by densitometry and represented as ratios of HIF-1α/β-tubulin and P-eIF2α/β-tubulin. Data were collected from 5 cell lines and expressed as mean ± SD. Correlation between HIF-1α and P-eIF2α was analysed by Pearson's correlation method. **P<0.01, γ=-0.943. (D) Pancreatic cancer cell lines (CFPAC-1 and BxPC-3) were treated with siHIF-1 and 2-ME and then evaluate the expression HIF-1α, CRT and P-eIF2α by Western blotting experiment.

We questioned whether the sensitization effect of PX-478 on ICD inducers is due to inhibition of HIF-1α. Induction of surface exposure of CRT (ecto-CRT) is one of the hallmarkers of ICD [28-30] and it occurs as a consequence of ER stress-induced phosphorylation of eIF2α [18, 19, 31]. Ecto-CRT was stained on non-permeabilized cells which had been treated individually as indicated. Gem or PX-478 alone had weaker effect on triggering CRT exposure but treatment with Gem/PX-478 showed strong induction of CRT surface exposure (Figure 4A). To determine the association between HIF-1α inhibition and eIF2α phosphorylation, treatment-induced alterations of HIF-1α, CRT, P-eIF2α and eIF2α protein expression were monitored in 5 PDAC cell lines. A typical ICD inducer, OXP (oxaliplatin) [18, 19] was used as a positive control. While none of these reagents induced changes in CRT and eIF2α expression, all of them mediated down-regulation of HIF-1α and up-regulation of P-eIF2α. Combination of PX-478 with either Gem or OXP led to maximum inhibition of HIF-1α and up-regulation of P-eIF2 α (Figure 4B). Treatment-induced changes in HIF-1α and P-eIF2α showed strong negative correlation, P<0.01, γ=-0.943 (Figure 4C). To further evaluate the effect of HIF-1 inhibition on ICD, PDAC cells were either transfected with HIF-1α-siRNA or treated with another HIF-1α inhibitor 2-Methoxyestradiol (2-ME) (Selleck. cn). As expected, both HIF-1α-siRNA and 2-ME induced eIF2α phosphorylation in PDAC cells (Figure 4D). These results demonstrate that inhibition of HIF-1α expression could trigger ICD by up-regulation P-eIF2α.

### Co-treatment with PX-478 enhances chemotherapy-induced HMGB1 and ATP release

**Figure 5:**
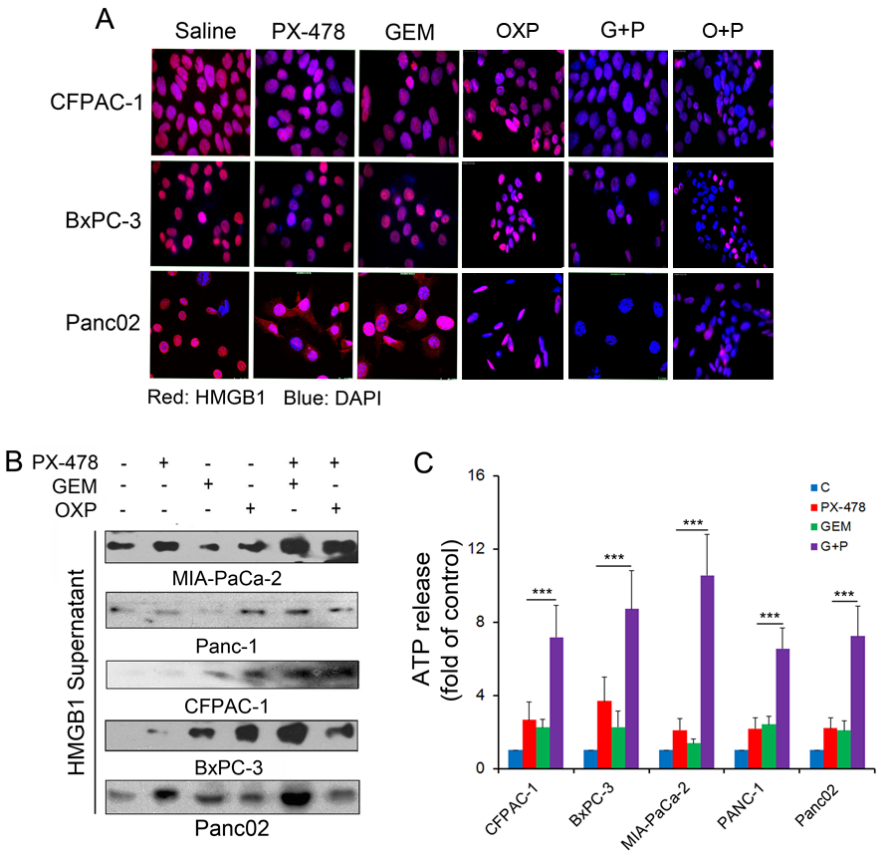
HMGB1 and ATP release in response to treatment PDAC cell lines were incubated with saline, Gem (1.0 μM), PX-478 (25 μM), or Gem/PX-478, OXP (oxaliplatin) (300μM) or OXP/PX-478 for 24 hours. (A) Determination of treatment-induced HMGB1 re-localization and release in three PDAC cell lines by fluorescent microscopy (magnification, 1000×). After treatment, cells on slides were fixed/permeablized and stained with both mouse anti-HMGB1 antibody/Alexa Fluor 568-conjugated secondary anti-mouse antibody (showing red) and DAPI (showing blue). (B) Determination of treatment-induced HMGB1 release in five PDAC cell lines by Western blotting. After treatment, 50 μl of conditioned medium was taken for detecting HMGB1 in the supernatants. (C) *Chemiluminescence* detection of treatment-induced ATP release in five PDAC cell lines. After treatment, 10 μl of conditioned medium was taken for ATP assay using chemiluminescence ELISA kit. Significantly increased ATP release by Gem/PX-478 (***P<0.001) was compared with those treated with single agent.

Release of HMGB1 and ATP from dying or dead cells are also crucial hallmarkers of ICD [18-20]. HMGB1 release from nucleus was determined by both immuno-fluorescent microscopy (Figure 5A) and Western blotting (Figure 5B). In control cells, HMGB1 is mainly expressed in the nucleus. Treatment with either Gem or PX-478 alone induced a translocation of HMGB1 from the nucleus to the cytoplasm. Co-treatment with Gem/PX-478 caused a loss of HMGB1 in the nucleus (Figure 5A). Western blotting confirmed that treatment with Gem/PX-478 induced maximum HMGB1 release compared with other treatment (Figure 5B). Similarly to HMGB1 release, Gem/PX-478 induced a significantly increased ATP release (P<0.001) in all 5 PDAC cell lines compared with other groups (Figure 5C). In summary, these data demonstrate that in the presence of HIF-1α inhibitor PX-478, GEM gains its ICD-inducing potential on triggering the exposure and release of ICD markers in pancreatic cancer cells.

### Conditioned medium or killed PDAC cells enhances immune response of DC and T cells

**Figure 6:**
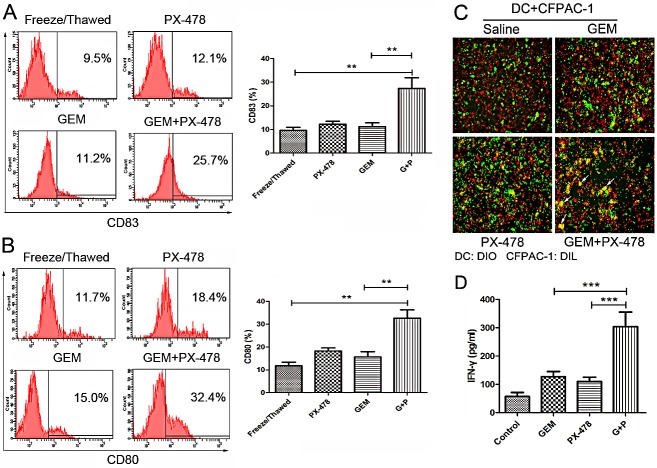
Comparison of treatment-induced immune response in both DCs and cytotoxic T cells (A and B) DC maturation. DCs were stimulated with conditioned supernatant of freeze/thawed, PX-478, Gem or Gem/PX-478 treated cell culture medium. The maturation markers for DCs, CD83 or CD80, were evaluated by flow cytometry and compared between freeze/thawed, PX-478, Gem and Gem/PX-478-treated groups. **P<0.01. Data were expressed as mean ± SD and each value represented the mean of three replicates. (C) Fluorescence microscopy analysis of phagocytosis. After 24 hours co-culture of immature DCs with killed tumor cells, the engulfment of tumor cells was verified by fluorescence microscopy. DCs were stained with DiO (green) and CFPAC-1 cells were stained with DiI (red). Double positive or yellow cells are fully phagocytized cells. (D) IFN-γ production. Immature DCs were pulsed with killed tumor cells for 24 hours and then co-cultured with autologous CD3^+^/CD8^+^ T lymphocytes. IFN-γ concentration in the supernatant was assessed after 10 days using the IFN-γ ELISA kit. A representative out of three independent experiments is depicted. ***P<0.001.

To test whether ICD markers in the conditioned medium could enhance immune response, human immature dendritic cells (iDCs) (treated with GM-CSF and IL-4 for 5 days) were incubated with conditioned supernatants from Gem or Gem/PX-478 treated cells for another 24 hours. Maturation of DCs was determined by expression of CD80 or CD83 using flow cytometry. Treatment with Gem/PX-478 significantly increased expression of both CD83 (Figure 6A) and CD80 (Figure 6B), indicating of maturation of DCs. To determine the phagocytosis single of ecto-CRT, iDCs (treated with GM-CSF and IL-4 for 5 days) were co-cultured with pancreatic cancer cells treated with saline, Gem, PX-478, or Gem/PX-478 for 24 hours. And then, fluorescence microscopy was used to evaluate the phagocytosis of DCs. DCs activated with Gem/PX-478-treated cells showed more phagocytosis activity compared with single treatment, as shown by double positive, or yellow coloured cells (Figure 6C). Most importantly, the co-culture Gem/PX-478 condition medium challenged DCs with autologous CD3^+^/CD8^+^ T cells significantly increased the secretion of IFN-γ by cytotoxic T-cells compared with control and those treated with single agent (Figure 6D). Taken together, these data confirmed that conditioned supernatants of Gem/PX478-treated PDAC cells can enhance immune response by inducing maturation and phagocytosis of DCs and up-regulate cytotoxic function of T cells.

## DISCUSSION

PDAC is highly resistant to both conventional and targeted chemotherapy. Increased numbers of circulating regulatory T cells, myeloid-derived suppressor cells and tumor-associated macrophages in the PDAC microenvironment suppress immune surveillance and dampen anti-tumor immune responses [32, 33]. Here we demonstrate, for the first time, that inhibition of HIF-1α expression by PX-478 enhances immunogenicity of Gem and elicits tumor-specific DC phagocytic and cytotoxic T cell responses to PDAC cells.

Although Gem is currently used as a first-line chemotherapeutic drug for advanced pancreatic cancer, it has only a 5.4% partial response rate and most patients do not respond well to single agent Gem and almost all the patients finally develop drug resistance [34]. Immunosuppression-induced by tumor microenvironment is one of the causes of failure in response to the treatment with Gem [33]. To promote the therapeutic effect of Gem, one rational approach is to combat drug-resistance by inducing ICD.

HIF-1α is an important transcription factor for tumor growth, invasion, angiogenesis, metabolism and drug resistance [7, 10, 25]. Typically, Gem-resistant pancreatic cancer cells overexpress HIF-1α that mediates acquired drug tolerance and tumor progression [12, 35]. Therefore, inhibition of HIF-1α would be a promising therapeutic strategy to strengthen the efficacy of Gem. We initially found that inhibition of HIF-1α by PX-478 increased the sensitivity of PDAC cells to Gem-induced apoptosis *in vitro* (data not show). Indeed, combination with PX-478 significantly increased inhibitory effect of Gem on tumor growth in PDAC-engrafted immune-competent mice. However, PX-478 did not improve the efficacy of Gem in an immune-deficient mice model. To confirm the immunogenic effect of this combination, we vaccinated treatment-induced dead cells to immune-competent mice and found that cells treated with Gem/PX-478 showed greater effect on eliminating tumor cells compared with vaccinated with cells treated with single agent. Most strikingly, more than half of mice after challenged with Gem/PX-478-treated cells reached long-term survival (60 days), indicating that the dead cell antigens-mediated by Gem/PX-478 can enhance immune surveillance and trigger anti-tumor immune response. As expected, we found significantly increased cytotoxic infiltrating T cells in both spleen and tumor tissues in Gem/PX-478-treated mice compared with those treated with single agent. PX-478 elicited immunogenicity on Gem was also confirmed by *in vitro* experiments. Conditioned medium from Gem/PX-478-treated PDAC cells triggered DC maturation and tumor-specific phagocytosis capacity and activation of tumor-specific cytotoxic T-cells but those treated with single agent showed minimum immunogenicity. These results indicate that either Gem or PX-478 is not ICD inducer but Gem obtained immunogenic potential when HIF-1α is inhibited.

We were interested in whether these treatment strategies could act differently in inducing ICD markers on tumor cells. Similar to immune responses, Gem/PX-478 showed higher potential in inducing CRT surface exposure, ATP and HMGB1 release compared with single treatment. It is known that CRT translocation from endoplasmic reticulum (ER) to the out-layer of plasma membrane depends on an ER stress and associated with ER kinase (PERK)-mediated eIF2α phosphorylation [18, 19]. We found that PX-478 induced HIF-1α inhibition is negatively correlated with eIF2α phosphorylation. To verify whether this effect on eIF2α phosphorylation is specifically due to inhibition of HIF-1α, we confirmed this phenomenon by using other HIF-1α inhibitor (2-ME) and HIF-1α-siRNA as well. Our results suggest that HIF-1α pathway might inhibit ER stress and subsequent CRT exposure. It has been reported that the ER stress is a consequence of production of reactive oxygen species (ROS) [36] and HIF-1α can decrease ROS production by reprogramming glucose metabolism [37]. We postulate that inhibition of ROS production by HIF-1α might be a possible mechanism to reduce ER stress. PX-478 also significantly enhanced Gem-induced ATP release. The function of ATP is to recruit monocytes and macrophages to the dying tumor cells and stimulate secretion of IL-1β, a key cytokine to polarize interferon-gamma-producing CD8+ T cells [38]. In addition, we observed reduction of HMGB1 from nuclear extracts of PX-478-treated cells and concomitant accumulation of HMGB1 in the conditioned medium. HMGB1 is a nuclear protein mediating stabilization of nucleosomes, DNA repair and recombination. Extracellular HMGB1 is required for the immunogenicity of ICD. It binds to the Toll-like receptor 4 (TLR-4) on the surface of DC and mediates cross-presentation efficiency [39]. We postulate that plasma HMGB1 concentration after treatment could also be used as a marker for predicting therapeutic response to Gem/PX-478 in patients with pancreatic cancer.

**Figure 7:**
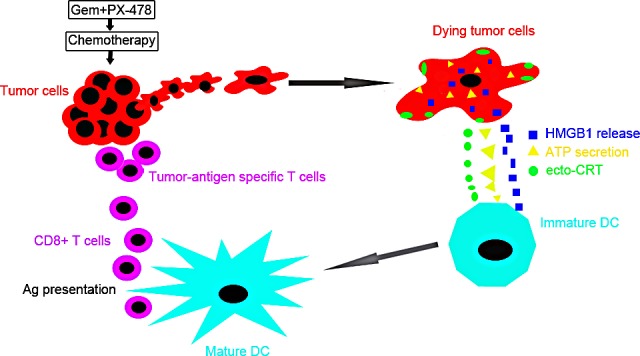
Combinatorial immunochemotherapy based on immunogenic cell death induced by Gem and PX-478 exerting synergistic anticancer activity Pancreatic cancer cells treated with Gem and PX-478 directly provoke an endoplasmic reticulum (ER) stress, leading to the release of damage-associated molecular patterns (DAMPs) within the tumor microenvironment (surface exposure of CRT (ecto-CRT), ATP secretion and HMGB1 release). DAMPs exposure leads to the specific recognition by antigen presenting cells (APCs), i.e., dendritic cells (DCs) and macrophages, which become mature and prime the tumor specific T and natural killer (NK) cells to react against tumor cell antigens. In sum, the combined administration of Gem and PX-478 might activate synergistic immunological response culminating in improved anticancer immune responses.

In recent years, new combination chemotherapeutic strategies gained better clinical benefit compared with single-agent Gem in pancreatic cancer. In 2011, the ACCORD-11/PRODIGE-4 trial (a multicenter randomized control phase III trial) confirmed that FOLFIRINOX (fluorouracil, leucovorin, irinotecan, and oxaliplatin) significantly prolonged the overall survival of patients with metastatic pancreatic cancer, compared with gem along (11.1 vs 6.8 months), although the safety profile of FOLFIRINOX was not as favourable as that of single-agent Gem [40]. In 2013, Gem plus nab-paclitaxel achieved better overall survival (median: 8.5 month vs 6.7 month) compared with Gem alone, based on a multinational phase III trial. Soon, the FDA approved Gem plus nab-paclitaxel as a first line treatment for patients with pancreatic cancer. Based on these achievements, combination of GEM and PX-478 provides a promising strategy to kill pancreatic cancer and need to be enrolled for clinical trials.

In conclusion, this study verified by both *in vivo* and *in vitro* experiments that combination of Gem with PX-478 effectively inhibited tumor growth by inducing ICD in pancreatic cancer. Gem/PX-478-mediated immunogenicity is dependent on eIF2α phosphorylation-associated CRT surface exposure, increased ATP and HMGB1 release. HIF-1α inhibition plays a key role in this sensitization on immune responses. Most importantly, we found that vaccination using Gem/PX-478-treated cells has great potential to prevent tumorigenesis.

## MATERIALS AND METHODS

### Cell lines, cell culture and *in vitro* treatment

Human PDAC cell lines CFPAC-1, BxPC-3, Panc-1, and MIA-PaCa-2 were obtained from the Cell Bank of Type Culture Collection of Chinese Academy of Sciences (Shanghai, China) and the American Type Culture Collection, respectively. The murine pancreatic cancer cell line Panc02 [41] was a gift from Prof. Yang SY. Cell lines were routinely cultured at 37 °C in a humidified atmosphere of 95% air and 5% CO_2_ using Dulbecco's modified Eagle medium (DMEM) with 10% fetal bovine serum (FBS). For *in vitro* treatment, 5 × 10^5^/ml cells in 6-well plate were treated with 1.0 μM Gem (Eli Lilly, Fegersheim, France) [24], or/and 25 μM PX-478 (MedKoo Biosciences, Inc, USA) [10, 42] for 24 hours in the routine culture condition. Cell death was determined by flow cytometry on Annexin-V/propidium iodide positive cells.

### Chemotherapy of established tumor in mice

All mice were maintained in specific pathogen-free conditions and the animal experiment procedures were approved by the Ethics Committee of Tianjin Medical University Cancer Institute and Hospital. Immuno-competent 4-week-old female C57BL/6 mice and immuno-incompetent nude (*Nu/Nu*) mice were injected subcutaneously in the flank with 1×10^6^ Panc02 cells. The tumor volumes were monitored every five days using digital callipers. When the tumor size reached 40-80mm^3^, mice were assigned into homogenous groups and treated with Gem (i.p at 15 mg/kg on days 1, 3, 5 every week) [6] or/and PX-478 (p.o. gavage at 30 mg/kg × 2 consecutive days) [42]. Tumor volumes were calculated using the formula π/6 [(short axis in mm)^2^ × (long axis in mm)] for comparison of tumor growth [43].

### Determination of peripheral and infiltrating CD3^+^ and CD8^+^ T lymphocytes

Tumor-bearing animals were euthanized by cervical dislocation as soon as they presented signs of necrosis. Peripheral blood was collected from the mice and spleen cell suspensions were produced by forcing the spleen through a wire mesh screen. Tumor single-cell suspension was prepared from tumor by digesting and mechanically dissociating with a MACS Dissociator (Miltenyi Biotec, Germany), followed by filtration through a 70-mm nylon mesh [44]. Cells were then co-stained with PE-anti-CD3 and APC-anti-CD8 antibodies (Biolegend, USA). Surface expression of CD3 and CD8 were determined by flow cytometry (BD FACS Canto II, BD, USA).

### Anti-tumor vaccination

A total of 3×10^6^ Panc02 cells, untreated or treated with either Gem and/or PX-478 for 24 hours, dying and dead cells/supernatant were inoculated subcutaneously into 6-week-old female C57BL/6 mice into the right flank, whereas 1×10^6^ untreated Panc02 cells were inoculated into the contralateral flank 7 days later [45, 46]. Animals that bore tumors in excess of 20–25% of the body mass or were necrotic, were euthanized.

### Western blotting

Whole-cell extracts were prepared by lysing cells with RIPA lysis buffer supplemented with protease inhibitor cocktails (Sigma). Protein concentrations were quantified using Pierce protein assay kit (Pierce). Cellular protein lysates (20 μg) were separated by 10% SDS-PAGE, and target proteins were detected by Western blotting using primary antibodies against CRT, HIF-1α, HMGB1, eIF2α, phosphorylated eIF2α (P-eIF2α), and β-tubulin (Suppl Table 1). Specific proteins were visualized using an enhanced chemiluminescence detection reagent (Pierce). For determining HMGB1 release, 50 μl of conditioned culture medium was loaded into each lane of SDS-PAGE gel and extracellular HMGB1 was detected by Western blotting using an anti-HMGB1 antibody [47].

### Preparation of human DCs and T lymphocytes

Human peripheral blood mononuclear cells (PBMCs) were isolated from healthy donors by Ficoll-Hypaque (Solarbio Co., China) density gradient centrifugation and cultured in RPMI 1640 medium containing 10% FBS for 2 hours. DCs were generated from the adherent fraction of PBMCs [48]. The adherent cells were cultured for 6 days in RPMI 1640 medium containing 10% FBS, 20 ng/mL human GM-CSF, and 10 ng/mL human IL-4 (Biolegend, USA). Culture medium and cytokines were refreshed every other day. On the day 6, the medium was replaced by the Gem or/and PX-478-treated conditioned medium or control medium and cell culture was continued for 24 hours. Autologous CD3+ T lymphocytes were magnetically isolated using CD3 MicroBeads (Miltenyi Biotech, Germany) to obtain purity ≥95% CD3+ T cells. The isolated T lymphocytes were cultured in RPMI 1640 medium containing 10% FBS and 10 ng/mL human IL-2 (Biolegend, USA), and the medium was replaced every other day.

### Immunostaining and fluorescent microscopy

The surface exposure of CRT was determined by immunostaining as previously described [28]. Briefly, after treatment cells were washed twice with PBS and fixed in 0.25% paraformaldehyde in PBS for 5 min. Cells were then washed with PBS for three times. The rabbit anti-calreticulin antibody (Cell Signaling Technology) was diluted in 3% BSA containing blocking buffer at 1:200 dilution and cells were stained with it overnight at 4°C. After washes in cold PBS, cells were stained with Alexa Fluor 488-conjugated secondary anti-rabbit antibody (Life technologies, USA) at 1:500 dilution for 30 min at room temperature. Nuclei were stained with Hoechst 33342 before images were viewed under OLYMPUS fluorescent microscope (Japan).

For determining intracellular HMGB1, cells on slides were fixed with 4% paraformaldehyde for 20 min and permeabilized with 0.1% Triton X-100 for 10 min. Nonspecific binding was blocked with 3% BSA in PBS for 30 min. Cells were stained with mouse anti-HMGB1 antibody (Sigma-Aldrich) at 1:100 dilution overnight at 4°C. Subsequently, cells were washed three times with PBS and incubated with Alexa Fluor 568-conjugated secondary anti-mouse antibody (Life technologies) for 30 min at room temperature in the dark [46]. Nuclei were stained with DAPI and HMGB1 localization was observed by fluorescent microscopy.

The anti-tumor phagocytic function of matured DCs was assessed after DCs or tumor cells were labelled fluorescently with *Vybrant^TM^* DiO (V-22886) or DiI (V-22885) cell-labelling solutions (Life technologies), respectively. Briefly, tumor cells were treated with Gem or/and PX-478 for 24 hours after stained with DiI (1:200 dilution). DiO-loaded immature DCs (Day 5) were then fed with treated tumor cells at a DC/tumor cell ratio of 5:1. After incubation for 24 hours, the cells were fixed with 4% paraformaldehyde for 20 min, washed in PBS for 20 min and mounted on slides [49] and viewed under a fluorescent microscope. Double positive cells were considered as fully phagocytosed cells.

### ATP release assay

ATP concentration in the culture medium was performed using an ATP Determination Kit (Life technologies) according to the manufacturer's instructions. Cell numbers were set up as 1 × 10^6^/ml for all experiments. After treatment for 24 hours, 10 μl of conditioned medium was taken out for ATP assay.

### IFN-γ production

IFN-γ secretion from CD3+ T lymphocytes was assessed by IFN-γ ELISA Kit (Uscn Life Science Inc, China) according to the manufacturer's instructions.

### RNA interference and cell transfection

Small interfering RNAs (siRNAs) against HIF-1α (Suppl Table 1) was designed and synthesized from GenePharma (Shanghai, China). Cells were transfected with HIF-1α-siRNA using Lipofectamine 2000 (Life Technologies) and knocking-down effect of HIF-1α was determined by Western blotting after 48 hours.

### Statistical analysis

Statistical analysis was performed using IBM SPSS version 18.0 for windows and GraphPad Prism software (version 5.03). For all *in vitro* experiments, at least 3 independent experiments were performed. For *in vivo* experiments, 5 to 8 mice were used for each group/per experiment. Data are expressed as mean ± SD. Significant difference between two groups with equal numbers were analysed by two-sided Student *t*-tests and those with unequal sizes were analysed with the Mann-Whitney *U* test. Two-way ANOVA with Bonferroni post-hoc test was used to compare restriction of tumor cell growth *in vivo* [50]. Kaplan-Meier survival curved were analyses using two-sided log-rank tests. Correlation between two groups of variables were analysed with Pearson correlation. All *P*-values <0.05 were considered as statistically significant.

## SUPPLEMENTARY MATERIAL TABLE AND FIGURE


