# Correction: Inhibition of HIF-1α by PX-478 enhances the anti-tumor effect of gemcitabine by inducing immunogenic cell death in pancreatic ductal adenocarcinoma

**DOI:** 10.18632/oncotarget.27205

**Published:** 2019-09-17

**Authors:** Tiansuo Zhao, He Ren, Li Jia, Jing Chen, Wen Xin, Fan Yan, Jing Li, Xiuchao Wang, Song Gao, Dong Qian, Chongbiao Huang, Jihui Hao

**Affiliations:** ^1^ Tianjin Medical University Cancer Institute and Hospital, National Clinical Research Center for Cancer, Key Laboratory of Cancer Prevention and Therapy, Department of Pancreatic Cancer, Tianjin, China; ^2^ Centre for Haemato-Oncology, Barts Cancer Institute, Queen Mary University of London, London, UK

**This article has been corrected:** Due to errors in image processing, there was an accidental duplication of data in Figure 4A. An updated Figure 4A using the original data is shown below. The authors declare that these corrections do not change the results or conclusions of this paper.

Original article: Oncotarget. 2015; 6:2250–2262. 2250-2262. https://doi.org/10.18632/oncotarget.2948

**Figure 4:** Negative correlation between HIF-1α expression and eIF2α phosphorylation. PDAC cell lines were incubated with saline, Gem (1.0 μM), PX-478 (25 μM), Gem/PX-478, OXP (oxaliplatin) (300μM) or OXP/PX-478 for 24 hours. (A) CRT surface exposure on CFPAC-1, BxPC-3 and Panc02 cell lines. Non-permeabilized cells were co-stained with anti-CRT antibody and Hoechst 33342 and determined by confocal microscopy (magnification, 600×). Green colour indicates ecto-CRT. (B) Five PDAC cell lines were treated with GEM, PX-478, OXP (300μM), Gem/PX-478, or Gem/OXP for 24 hours. Expression of HIF-1α, CRT, P-eIF2α, and eIF2α was determined by Western blotting. β-tubulin was used as a loading control and OXP was served as a positive control. (C) Negative correlation between levels of HIF-1α and P-eIF2α. Expression levels of both HIF-1α and P-eIF2α were analysed by densitometry and represented as ratios of HIF-1α/β-tubulin and P-eIF2α/β-tubulin. Data were collected from 5 cell lines and expressed as mean ± SD. Correlation between HIF-1α and P-eIF2α was analysed by Pearson’s correlation method. **P<0.01, γ=-0.943. (D) Pancreatic cancer cell lines (CFPAC-1 and BxPC-3) were treated with siHIF-1 and 2-ME and then evaluate the expression HIF-1α, CRT and P-eIF2α by Western blotting experiment.

