# Supplementary material for: Inhibition of HIF-1α by PX-478 enhances the anti-tumor effect of gemcitabine by inducing immunogenic cell death in pancreatic ductal adenocarcinoma
Source: Oncotarget. 2014 Dec 10;6(4):2250–62. doi: 10.18632/oncotarget.2948 (PMC4385849; doi:10.18632/oncotarget.2948)
Supplement: Supplementary file 1 [file oncotarget-06-2250-s001.pdf]

Inhibition of HIF-1α by PX-478 enhances the anti-tumor effect of gemcitabine by inducing immunogenic cell death in pancreatic ductal adenocarcinoma

Supplementary Material

Supplementary Table 1. Antibodies and siRNA sequences

**Antibodies**				
Name	Manufacturer	Number	Type	Usage
HIF-1α	Abcam	ab113642	Monoclonal	WB
CRT	Cell Signaling Technology	12238	Polyclonal	WB,IF
eIF2α	Cell Signaling Technology	9722	Polyclonal	WB
P-eIF2α	Cell Signaling Technology	9721	Polyclonal	WB
HMGB1	Sigma	WH0003146M8	Monoclonal	WB.IF
β-tubulin	Abmart	M30109	Monoclonal	WB
**siRNAs**	**sequences (5'-3')**			
HIF-1α #1	CCAGCAGACUCAAAUACAATT/	UUGUAUUUGAGUCUGCUGGTT		
HIF-1α #2	GCCUCUUUGACAAACUUAATT/	UUAAGUUUGUCAAAGAGGCTT		
HIF-1α #3	CAGGCCACAUUCACGUAUATT/	UAUACGUGAAUGUGGCCUGTT		

WB: Western boltting; IF:Immunofluorescence

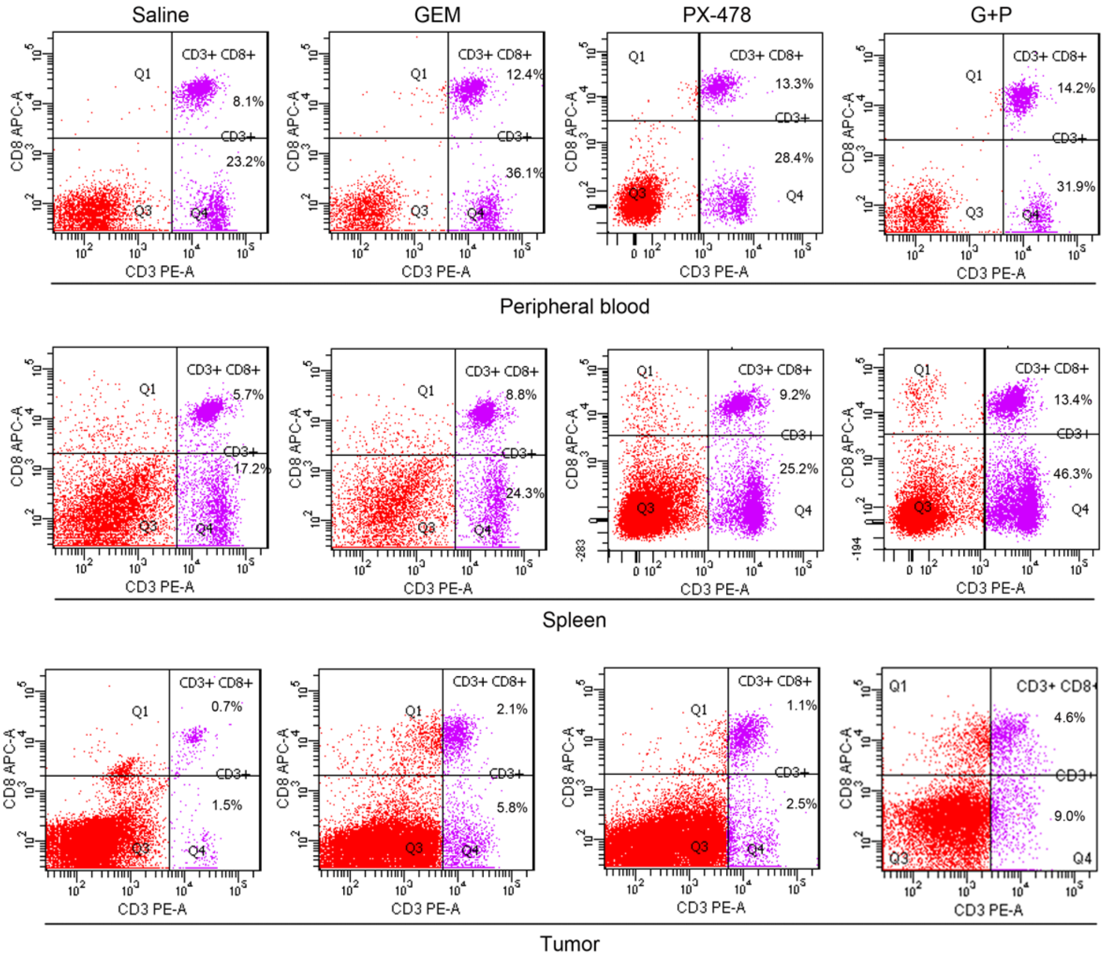

Supplementary figure1: The figures of $CD8^{+}$ and $CD3^{+}$ T cells isolated from peripheral blood, spleen and tumour were analyzed by flow cytometry.
